# Construction and experimental validation of a B cell-related gene signature to predict the prognosis and immunotherapeutic sensitivity in bladder cancer

**DOI:** 10.18632/aging.204753

**Published:** 2023-06-27

**Authors:** Ranran Zhou, Jiawei Zhou, Bahaerguli Muhuitijiang, Xiangbo Zeng, Wanlong Tan

**Affiliations:** 1 Department of Urology, Nanfang Hospital, Southern Medical University, Guangzhou 510000, Guangdong, China

**Keywords:** B cell, bladder cancer, prognosis, immunotherapy, tumor immunity

## Abstract

Background: B cells are essential components of tumor microenvironment and exert important functions in anti-tumor immune response. However, the prognosis value of B cell-related genes in bladder cancer (BLCA) remains obscure.

Materials and Methods: The infiltrating levels of B cells were measured via the CD20 staining in the local samples and the computational biology analyses in the TCGA-BLCA cohort. The single-cell RNA sequencing analysis, gene-pair strategy, LASSO regression, random forest, and Cox regression were used for B cell-related signature construction. TCGA-BLCA cohort was chosen as the training cohort, and three independent cohorts from GEO and the local cohort were used for external validation. 326 B cells were adopted to explore the association between the model and B cells’ biological processes. TIDE algorithm and two BLCA cohorts receiving anti-PD1/PDL1 treatment were utilized to detect its predictive ability to the immunotherapeutic response.

Results: High infiltration levels of B cells heralded favorable prognosis, both in the TCGA-BLCA cohort and the local cohort (all P < 0.05). A 5-gene-pair model was established and served as a significant prognosis predictor across multiple cohorts (pooled hazard ratio = 2.79, 95% confidence interval = 2.22-3.49). The model could evaluate the prognosis effectively in 21 of 33 cancer types (P < 0.05). The signature was negatively associated with B cells’ activation, proliferation, and infiltrating levels, and could serve as a potential predictor of immunotherapeutic outcomes.

Conclusions: A B cell-related gene signature was constructed to predict the prognosis and immunotherapeutic sensitivity in BLCA, helping to guide the personalized treatment.

## INTRODUCTION

As one of the most common malignancies worldwide, with about 500,000 new cases annually, bladder cancer (BLCA) is characterized by high morbidity and mortality and poor prognosis [1]. Although many different treatments, such as transurethral resection of bladder tumor, intravesical therapy with Bacille Calmette–Guérin, and neoadjuvant chemotherapy, have been widely adopted in clinical practice, the prognosis of BLCA is still far from satisfactory [2]. In recent decades, the proposal and application of immunotherapy, represented by immune-checkpoint inhibitors (ICIs), and targeted therapy, such as erdafitinib, an FGFR kinase inhibitor, have raised new hopes to cure this disease [3, 4]. Unfortunately, while these therapeutic advances have favorably altered many patients’ clinical outcomes, the benefits are still limited to a fraction of the cases. Therefore, the mainstay challenges lying ahead include the seeking of more drug targets to improve the prognosis and the development of reliable and robust biomarkers to evaluate the clinical outcomes, which are also the aims of the present study.

The tumor microenvironment (TME) is a complex ecosystem involving various cancer cells, immune cells, and stromal cells, and these cells interact and mutually regulate with each other, suppressing or promoting tumor growth [5]. Besides, as mentioned above, most cases with solid cancer cannot receive benefits from ICIs, which are based on the functions of T cells. These facts enlighten researchers to study the immune regulative functions of other immune cells in TME, among which B cells have attracted increasing attention. A meta-analysis containing 69 studies representing 19 cancer types revealed that the higher level of tumor-infiltrating B cells heralded favorable prognosis in most cancers, suggesting that B cells play a nonnegligible role in tumor development [6]. Mechanistically, after B cells differentiate into plasma cells, they could directly kill tumor cells by antibody-dependent cell-mediated cytotoxicity, complement-dependent cytotoxicity, antibody-dependent cellular phagocytosis, or other means [7]. B cells also promote the formation of tertiary lymphoid structures, enhancing the activation of T cells [8]. At the same time, B cells could possess pro-tumor functions, which are mainly represented by B regulatory cells (Bregs). Bregs refer to a cluster of B cells secreting interleukin-10 (IL-10) and contributing to immunosuppression [9]. In view of the crucial roles B cells play in tumorigenesis and tumor immunity, the treatment targeting B cells might be a promising strategy. However, first, comprehensive analyses of the biomarkers in B cells and their association with the prognosis and immunotherapeutic sensitivity are urgently needed.

Herein, the present study explored the prognosis value of B cell infiltrating levels in the local cohort and the TCGA-BLCA cohort at first. Next, a B cell-related gene model was established to evaluate the prognosis of BLCA patients. The B cells’ marker genes were identified from the single-cell RNA sequencing (scRNA-seq) of a BLCA sample. The samples from the Cancer Genome Atlas (TCGA) were chosen as the training cohort. To render the predictive model applicable in different gene detection platforms, we adopted a gene-pair strategy, as previously described [10]. Various machine learning algorithms, including LASSO regression and random forest, were used for feature selection. Gene Expression Omnibus (GEO) was comprehensively queried, and three cohorts were selected for external validation. We also collected 19 BLCA samples, along with their clinicopathological features and follow-up information, from the local hospital to reconfirm the association between the established model and BLCA’s prognosis. 326 B cell samples extracted from the scRNASeqDB database were used to investigate the association of BCRS with B cells’ biological processes. TIDE algorithm, IMvigor210 cohort, and GSE111636 cohort were used to investigate the predictive ability of the model to immunotherapeutic sensitivity. We also conduct the pan-cancer analysis of the established model to investigate its clinical usefulness in other cancer types.

## MATERIALS AND METHODS

### Data collection and processing

The transcriptome sequencing of 19 porocarcinoma and 411 BLCA samples in “count” and “FPKM” format, along with the corresponding clinical and follow-up information, was obtained from TCGA (https://portal.gdc.cancer.gov/), as the training dataset. The gene expression matrices with the “count” format and the “FPKM” format were utilized for gene expression difference detection and prognostic model construction, respectively. We queried GEO (https://www.ncbi.nlm.nih.gov/geo/) to download the external validation datasets using the keyword “bladder cancer.” The datasets would be included if they met the following criteria: (1) The transcriptome sequencing data of the patients with BLCA was publicly available. (2) The survival statuses and follow-up duration or their clinical response to immunotherapy can be downloaded in the GEO database in the Supplementary Materials of the original article. (3) The expressions of the genes in the established model must be included in the dataset. Two researchers (Zhou R and Zhou J) independently extracted the dataset and reached a consensus for all items, and any conflicts that arose were resolved with an expert (Tan W) invited to join the discussion.

After the careful and manual review of retrieval results in the GEO, four datasets, including GSE13507 [11, 12], GSE31684 [13, 14], GSE32894 [15], and GSE111636, were extracted. The detailed information on these GEO datasets is displayed in Supplementary Table 1. Given that the GSE111636 dataset, which included 6 responders and 5 non-responders to pembrolizumab, has not been publicly published, the IMvigor210 cohort, containing 195 BLCA samples, was used in this study, whose transcription data and clinical information can be obtained from the IMvigor210CoreBiologies package in R software (version 4.0.3) [16]. In these cohorts, the cases with less than 30 days’ follow-up and the gene with average expression values < 0.5 were excluded.

### Clinical sample collection

The tumor and the adjacent normal tissues from 19 BLCA patients undergoing partial/radical cystectomy without prior radiotherapy and chemotherapy were collected from the Nanfang Hospital of Southern Medical University (Guangzhou, China) between 2020 and 2022, which was named “NH cohort.” The BLCA samples were isolated from the center of the tumor and immediately stored in liquid nitrogen. The formalin-fixed paraffin-embedded BLCA tissue specimen was kindly provided by the Department of Pathology of Nanfang Hospital. The diagnosis of the tumor-node-metastasis (TNM) stages of the BLCA samples depended on the eighth TNM staging system defined by the American Joint Commission on Cancer.

### Identification of B cell marker genes

Compared with other public scRNA-seq datasets extracted from GEO, such as GSE135337 [17] and GSE190888 [18], the GSE145137 [19] dataset contained a higher infiltrating ambulance of B cells and was chosen for the B cell markers’ identification. Here, to avoid latent interference, we only used the tumor sample isolated from a patient with primary BLCA (GSM4307111) to conduct the analyses, and a total of 2075 cell samples were included. The Seurat package of R software was utilized to process the scRNA-seq data. Principal component analysis (PCA) and t-distributed stochastic neighbor embedding (t-SNE) were conducted to divide the cell samples into various cell clusters. The cell marker genes with identified by the “FindAllMarkers” function of the Seurat package, and the filtering thresholds were set as follows: | logarithmic fold change [logFC]| > 0.5, adjusted P value < 0.05, and pct > 0.25, where pct represented the proportion of a gene with non-zero values in a cell cluster. At last, the SingleR package in R was utilized to perform the cell type annotation.

### B cells’ infiltrating profiles identified by computational biology and TIDE algorithm

Different publicly available algorithms, including xCELL, TIMER, quanTIseq, MCPCOUNTER, and EPIC, were employed to estimate the B cells’ infiltration proportion. TIDE algorithm, aiming to predict the immunotherapeutic response based on the transcriptome profiles of cancer samples, was adopted to partly verify the predictive ability of the established model to immunotherapeutic sensitivity [20].

### Genomic difference analysis, gene functional annotation, and protein-protein interaction (PPI) network construction

The edgeR package was implemented to identify the differentially expressed genes between paracarinoma and BLCA tissues with |logFC| > 0.5 and false discovery rate (FDR) < 0.05 filtering. The functional annotation of the screened genes was performed through the Metascape (https://metascape.org) with default settings. The PPI network was constructed in the STRING database (https://cn.string-db.org) with a confidence level = 0.4.

### Meta-analyses

The meta-analyses were conducted to pool the hazard ratios (HRs) through the meta package in R. As a general rule, if the heterogeneity test was not significant (I^2^ >50% and P value < 0.05), the random effect model would be used to generate the summary effect size of studies; otherwise (I^2^ <50% and P value > 0.05), the fixed effect model would be employed to combine the effect values.

### Risk model construction

The genes con-determined by the B cell marker genes’ identification and the genomic divergence analysis between adjacent normal and tumor tissues were chosen for further study. As previously described [21], a gene-pair strategy was employed to construct numerous gene pairs to render the predictive model applicable in different gene expression detection platforms. Briefly, “gene A|gene B” represented a gene pair, and this combination would be regarded as 1 if the expression of gene A is higher than that of gene B; conversely, it would be considered as 0. LASSO regression with 10-fold cross-validation and random forest with nsplit at 10 at the variable hunting was used to identify the significant gene pairs associated with the overall survival (OS) through the glmnet and the randomforestSRC packages, respectively. In the random forest analysis, the VIMP of each variable was calculated, which was positively associated with the variable importance, and the pair with VIMP > 0.001 was chosen. At last, the predictive model was constructed via the multivariate Cox regression with stepwise, and the risk score, named B cell-related score (BCRS), of each sample was calculated. The BCRS was constructed according to the following formula: $\text{BCRS}=\sum_{\text{i}=1}^{\text{n}}{\text{Coeff}}_{\text{i}}*{\left(\text{gene}\;\text{pair}\right)}_{\text{i}}$. The optimal cut-off value to divide the patients in the TCGA-BLCA cohort into a high- or low-BCRS subgroup was detected by the X-tile, and the cut-off was then applied in all the cohorts enrolled in this study. The survival package in R was used to conduct the Kaplan-Meier survival analyses with log-rank tests. The nomogram to visualize the predictive model was drawn by the rms package.

### Pan-cancer analyses

The transcriptome profiles and their corresponding survival information of the other 32 cancer types in the TCGA database were obtained from the UCSC Xena website (https://xenabrowser.net/datapages/). The survival information included disease-free interval (DFI), disease-specific survival (DFS), OS, and progression-free interval (PFI), and the cases with less than 30 days’ follow-up were excluded.

### Gene set enrichment analysis (GSEA)

GSEA was performed based on the GSEA software (version 4.3.2), which was directly downloaded from the GSEA official website (http://www.gsea-msigdb.org/gsea/). The reference gene sets about the biological processes of B cells were obtained from the Molecular Signature Database (http://www.gsea-msigdb.org/gsea/msigdb/). The terms with Nominal P < 0.05 and FDR < 0.25 were considered to be statistically significant.

### Cell culture

The human normal urethral epithelial cell line, SVHUC1, and two human BLCA cell lines, including T24, and UMUC3, were purchased from the Type Culture Collection of the Chinese Academy of Sciences (Shanghai, China). SVHUC1 cells were maintained in Ham's F-12K Media (Invitrogen, USA). T24 and UMUC3 cells were incubated in Dulbecco’s Modified Eagle Medium (Invitrogen, USA). Those media were supplemented with 10% FBS and 1% antibiotics, and all of the cells were cultured in a humidified atmosphere with 5% CO2 at 37° C.

### Real-time quantitative PCR (RT-qPCR)

Trizol reagent (ThermoFisher Scientific, Germany) was used to extract the total RNA of the BLCA and paracarinoma tissue. PrimeScript RT Reagent Kit (Takara, China) and SYBR Premix ExTaq kit (Takara, China) were utilized for cDNA synthesis and amplification. GAPDH was chosen as the internal reference gene, and the detected expression values were normalized via the 2-ΔΔCt based on the ABI Prism 7000 system (Applied Biosystems, USA). The primer sequence used is shown in Supplementary Table 2.

### Immunohistochemical staining (IHC) and B cell counting

IHC was conducted in the BLCA specimen using the monoclonal antibody against CD20 (dilution: 1:100, ABclonal, China). The B cells in the slides were distinguished from other cells by staining of CD20, as reported by previous studies [22, 23]. The detailed processes of IHC in the BLCA tissue slides were well described in our earlier study [24]. Each sample was assessed under a microscope at x200 magnification, and the average B cell number was evaluated based on 5 areas with the highest number of CD20+ cells.

### Statistical analyses

The statistical analyses of the whole study were conducted via the R software (version 4.0.3) and GraphPad Prism (version 8.0.1). Unless otherwise specified, Wilcoxon signed-rank test was adopted for the continuous variables in two groups, and the Kruskal-Wallis test would be utilized if the number of groups ≥ 3. Pearson’s chi-square test and Fisher’s exact test were performed to measure the divergence for the categorical variables. The Spearman-correlation calculation was performed using the “cor.test” function in R. Welch’s corrected t-test was used to compare the results from the RT-qPCR and B cell counting experiments. The time-dependent receiver operating curve (ROC) analyses were performed and areas under curves (AUCs) were calculated by the timeROC package. In this study, P value < 0.05 was considered significant.

### Data availability

The data used to develop the predictive model was downloaded from the TCGA database (https://portal.gdc.cancer.gov/). The validation datasets, including GSE13507, GSE31684, GSE32894, GSE111636, and GSE145137, were obtained in the GEO website (https://ncbi.nlm.nih.gov/geo/). The IMvigor210 dataset was extracted in the IMvigor210CoreBiologies package in R, which could be downloaded from the official website (https://www.r-project.org/). The R code would be provided by the corresponding author upon reasonable request.

## RESULTS

### Computational biology indicated B cells’ infiltrating was associated with prognosis and immunotherapeutic outcomes in BLCA

**Figure 1:**
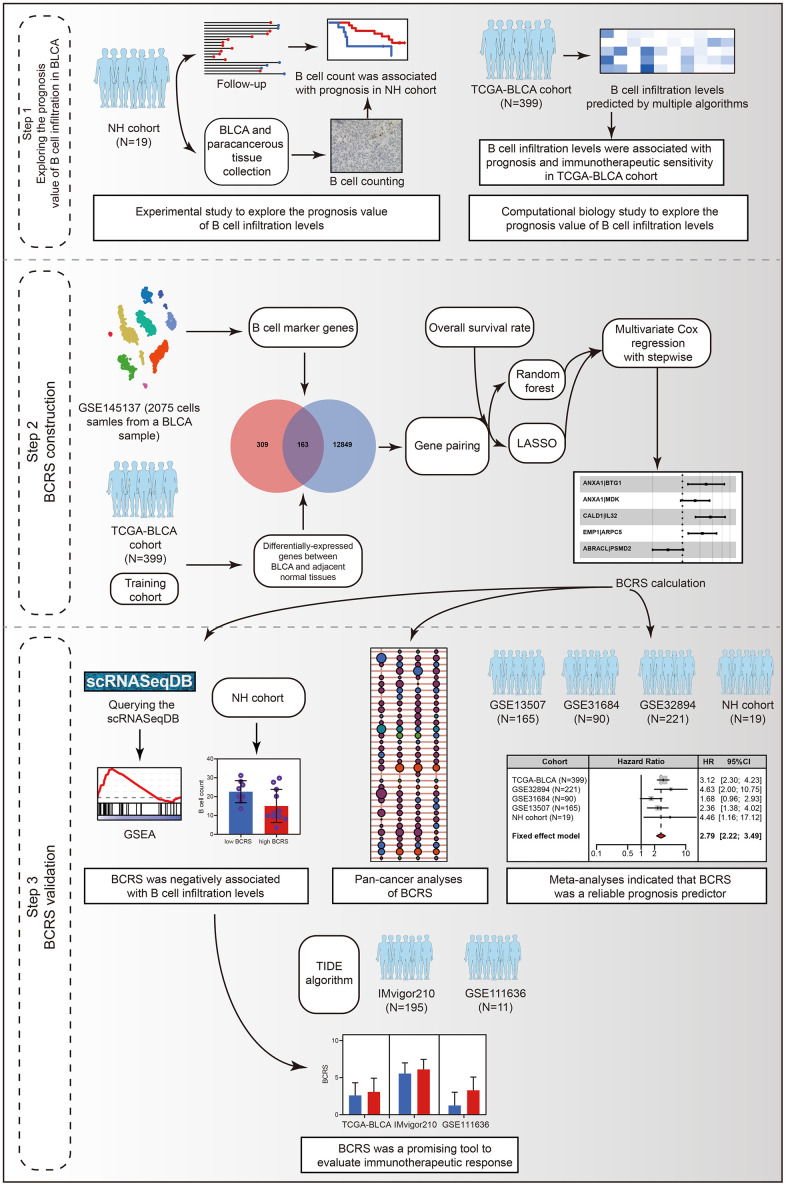
The workflow of the present study.

**Figure 2:**
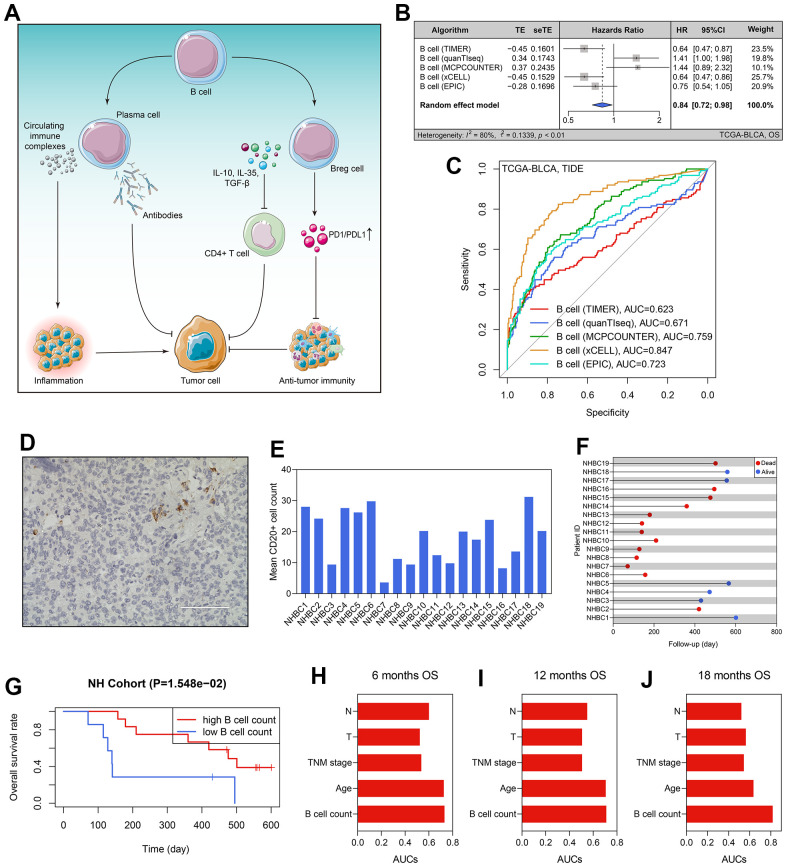
**B cells’ prognosis value in BLCA.** (**A**) The dual roles of B cells in tumor immunity. (**B**) The B cells’ infiltration level was a predictor for OS in the TCGA-BLCA cohort. (**C**) The predictive ability of B cells’ infiltrating proportion to immunotherapeutic sensitivity. (**D**) B cell number in each BLCA specimen was quantified using CD20 staining. (**E**) The B cell counts of the patients form the NH cohort. (**F**) The survival statuses and follow-up duration of the patients in the NH cohort. (**G**) High infiltration level of B cells heralded favorable prognosis in the NH cohort. (**H**–**J**) The comparison of the predictive ability of B cell number and routine clinicopathological traits to 6- (**H**), 12- (**I**), and 12-months’ OS (**J**). BLCA, bladder cancer; OS, overall survival.

The workflow of the whole study is graphically presented in Figure 1. As described above, B cells play a dual role in tumorigenesis. On the one hand, B cells can kill the tumor cells by secreting antibodies and inducing a series of anti-tumor immune processes; on the other hand, B cells also have a pro-tumor effect, which was mainly contributed by the Breg cells and circulating immune complexes secreted by plasma cells [6] (Figure 2A). Besides, despite the fact that the previous studies have disclosed the prognosis value of B cells in 19 cancers, it remains unclear in BLCA [6]. Hence, it is demanded and meaningful to clarify the predictive ability of B cells to clinical outcomes in BLCA at the initial stage of this research. The B cells’ infiltrating profiles were evaluated by multiple publicly available algorithms, including xCELL, TIMER, quanTIseq, MCPCOUNTER, and EPIC, where CIBERSORT-ABS and CIBERSORT were not adopted because they only analyzed the B cells’ subtypes. The infiltration levels in each BLCA sample in the TCGA-BLCA cohort are shown in Supplementary Table 3. The meta-analysis indicated that the subjects with high B cells’ infiltration exhibited a favorable OS rate (pooled HR = 0.84, 95% confidence interval [CI] = 0.72-0.98, Figure 2B). After evaluating the immunotherapeutic response through TIDE, we observed that B cell infiltration was a promising predictor for immunotherapeutic sensitivity (Figure 2C). It should be emphasized that the conclusion was drawn by *in silico* analysis, though it has been verified in many other cancers [6].

### High infiltrating of B cells heralded favorable prognosis in the NH cohort

To better confirm the prognostic value of B cell infiltrating levels, we collected the tumor samples of 19 BLCA patients from the Nanfang Hospital of Southern Medical University, and then performed the CD20 staining to quantify the B cell number in each specimen (Figure 2D). The clinicopathological features of these patients were extracted from the local hospital’s electronic medical record system (Table 1), and the average B cell number of each BLCA sample is displayed in Figure 2E. Additionally, we also recorded the follow-up data and survival statuses of these 19 BLCA patients (Figure 2F). According to the optimal cut-off value detected by the X-tile software, 19 BLCA patients were divided into high- and low-B cell count subgroups, and the patients with lower B cell infiltration levels suffered poorer OS (P < 0.05, Figure 2G). Compared with routine clinicopathological parameters, including age, TNM stages, pathological T stages, and pathological N stages, B cell number in the BLCA samples was of a stronger predictive ability to prognosis, no matter for 6- (Figure 2H), 12- (Figure 2I), or 18-months’ OS (Figure 2J).

**Table 1:** The baseline clinical information of the BLCA patients.

**Parameters**	**TCGA-BLCA (n=399)**	**GSE13507 (n=165)**	**GSE31684 (n=90)**	**GSE32894 (n=221)**	**NH cohort (n=19)**
**Survival status**					
Alive	223 (55.89%)	96 (58.18%)	27 (30.00%)	196 (88.69%)	6 (31.58%)
Dead	176 (44.11%)	69 (41.82%)	63 (70.00%)	25 (11.31%)	13 (68.42%)
**Follow-up (days)**	825.53 ± 835.05	1451.45 ± 1131.13	1471.24 ± 1339.92	1213.05 ± 759.96	346.16 ±188.22
**Age (years)**	68.01 ± 10.66	65.18 ± 11.97	69.00 ± 10.18	69.41 ± 11.25	65.42 ± 10.52
**Gender**					
Female	106 (26.57%)	30 (18.18%)	24 (26.67%)	60 (27.15%)	1 (5.26%)
Male	293 (73.43%)	135 (81.82%)	66 (73.33%)	161 (72.85%)	18 (94.74%)
**TNM Stage**					
0a	0 (0.00%)	23 (13.94%)	-	-	0 (0.00%)
I	2 (0.50%)	80 (48.48%)	-	-	6 (31.58%)
II	125 (31.33%)	26 (15.76%)	-	-	5 (26.32%)
III	138 (34.59%)	21 (12.73%)	-	-	6 (31.58%)
IV	132 (33.08%)	15 (9.09%)	-	-	2 (10.53%)
Unknown	2 (0.50%)	0 (0.00%)	-	-	0 (0.00%)
**pT stage**					
Ta	0 (0.00%)	24 (14.55%)	5 (5.56%)	109 (49.32%)	0 (0.00%)
T1	4 (1.00%)	80 (48.48%)	10 (11.11%)	61 (27.60%)	6 (31.58%)
T2	114 (28.57%)	31 (18.79%)	17 (18.89%)	43 (19.46%)	5 (26.32%)
T3	191 (47.87%)	19 (11.52%)	40 (44.44%)	7 (3.17%)	4 (21.05%)
T4	58 (14.54%)	11 (6.67%)	18 (20.00%)	1 (0.45%)	4 (21.05%)
Unknown	32 (8.02%)	0 (0.00%)	0 (0.00%)	0 (0.00%)	0 (0.00%)
**M stage**					
M0	189 (47.37%)	158 (95.76%)	56 (62.22%)	-	19 (100.00%)
M1	10 (2.51%)	7 (4.24%)	34 (37.78%)	-	0 (0.00%)
Unknown	200 (50.13%)	0 (0.00%)	0 (0.00%)	-	0 (0.00%)
**pN stage**					
N0	230 (57.64%)	149 (90.3%)	-	27 (12.22%)	14 (73.68%)
N1	46 (11.53%)	8 (4.85%)	-	3 (1.36%)	4 (21.05%)
N2	75 (18.80%)	6 (3.64%)	-	10 (4.52%)	1 (5.26%)
N3	7 (1.75%)	1 (0.61%)	-	0 (0.00%)	0 (0.00%)
Unknown	41 (10.28%)	1 (0.61%)	-	181 (81.9%)	0 (0.00%)
**Grade**					
High	378 (94.74%)	60 (36.36%)	84 (93.33%)	-	19 (100.00%)
Low	18 (4.51%)	105 (63.64%)	6 (6.67%)	-	0 (0.00%)
Unknown	3 (0.75%)	0 (0.00%)	0 (0.00%)	-	0 (0.00%)
**Risk stratification**					
High (> 2.75)	170 (42.61%)	42 (25.45%)	23 (25.56%)	71 (32.13%)	11 (57.89%)
Low (≤ 2.75)	229 (57.39%)	123 (74.55%)	67 (74.44%)	150 (67.87%)	8 (42.11%)
**BCRS**	2.93 ± 1.81	2.37 ± 1.17	2.12 ± 1.05	2.61 ± 1.27	4.00 ± 2.28

BCRS, B cell-related score; BLCA, bladder cancer.

### Construction of BCRS

**Figure 3:**
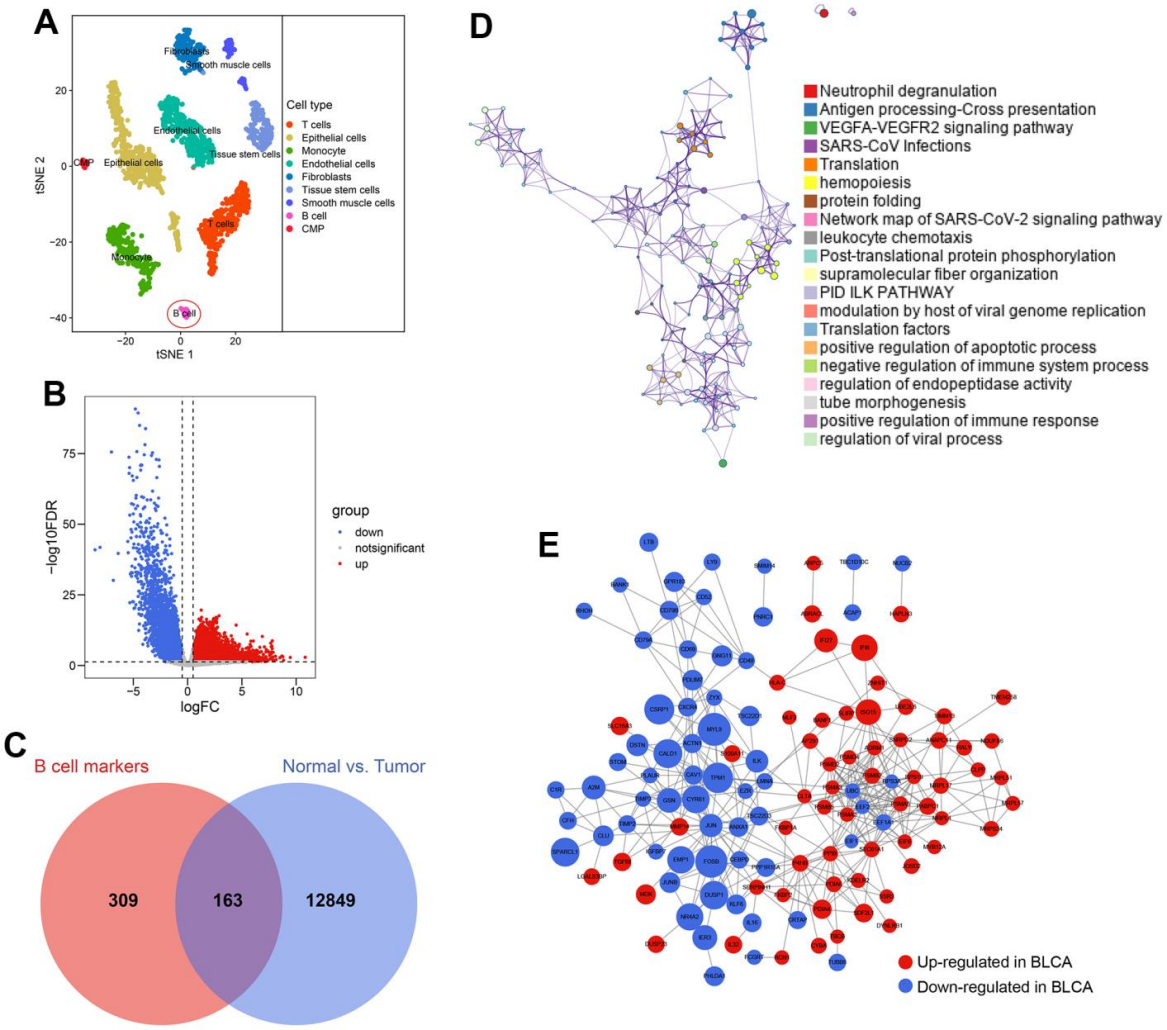
**Identification of B cell-related genes.** (**A**) 2075 cells from the primary BLCA were divided into 9 different cell types. (**B**) 13012 genes showing transcriptome expression difference between paracarinoma and BLCA samples were identified in the TCGA-BLCA cohort. (**C**) 163 genes were con-determined by the B cell marker genes’ analyses and genomic difference analyses. (**D**) The functional annotation of the 163 genes. (**E**) The protein-protein interaction network of the 163 genes.

2075 cell samples extracted from a BLCA patient were mainly divided into 9 cell types, which are displayed in Figure 3A. A sum of 472 cell marker genes of B cells was identified, which were differentially expressed in B cells compared with other cell types (Supplementary Table 4). At the same time, 13012 differentially expressed genes between the paracarinoma and BLCA samples were screened from the TCGA-BLCA cohort (Figure 3B and Supplementary Table 5), 163 of which also acted as B cell markers and were selected for further investigation (Figure 3C). The functional annotation indicated that the 163 genes were primarily involved in the immune and inflammatory responses (Figure 3D), agreeing with the previous findings. The PPI network of these 163 genes is shown in Figure 3E, implying the tight regulatory relationship of these genes.

**Figure 4:**
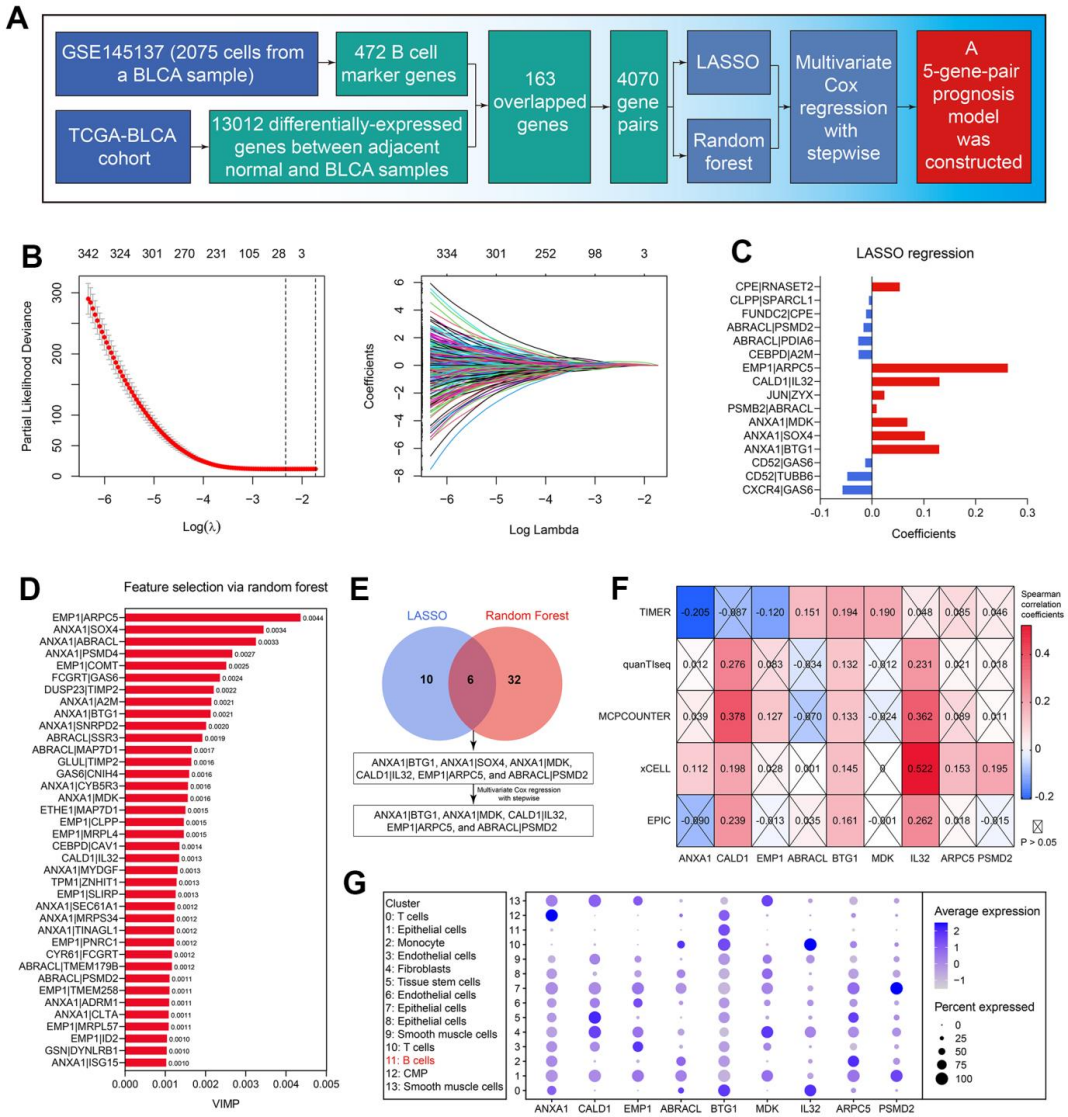
**Establishment of the B cell-related gene signature.** (**A**) The workflow showed the process of the predictive model construction. (**B**) The LASSO regression identified 16 gene-pairs significantly associated with OS in the TCGA-BLCA cohort. (**C**) The coefficients of the variables in the LASSO regression model. (**D**) The random forest algorithm identified 38 gene-pairs with VIMP > 0.001. (**E**) 6 gene-pairs were con-determined by the LASSO and random forest analyses, 5 of which were identified with the multivariate Cox regression with stepwise. (**F**) The association of the genes in the predictive model with B cells’ infiltration proportion. (**G**) The expression levels of the genes in the established model in each cell cluster.

Subsequently, the 163 genes were cyclically singly paired, and 4070 gene pairs were constructed. The workflow of the predictive model construction is graphically displayed in Figure 4A. First, LASSO regression identified 16 gene pairs as the significant predictor for OS in the TCGA-BLCA cohort (Figure 4B), and their coefficients were shown in Figure 4C. Meanwhile, in the random forest analysis, 38 gene pairs were determined (Figure 4D and Supplementary Table 6). 6 gene pairs intersected with the LASSO regression results and random forest outcomes were then included in the multivariate Cox regression with stepwise (Figure 4E). Accordingly, the predictive model was constructed. BCRS was calculated as follows: BCRS = 0.548*(ANXA1|BTG1) + 0.294*(ANXA1|MDK) + 0.645*(CALD1|IL32) + 0.461*(EMP1|ARPC5) - 0.334*(ABRACL|PSMD2). A sum of 9 genes was contained in this predictive model, including ANXA1, CALD1, EMP1, ABRACL, BTG1, MDK, IL32, ARPC5, and PSMD2, almost all of which were significantly associated with B cell infiltration proportion (all P < 0.05, Figure 4F), indicating that BCRS might be associated with B cell infiltration in BLCA samples (see below for more analyses). Figure 4G shows the expression levels of these 9 genes in each cell cluster identified by the scRNA-seq analysis of the BLCA sample.

### BCRS was a robust prognosis predictor for BLCA

**Figure 5:**
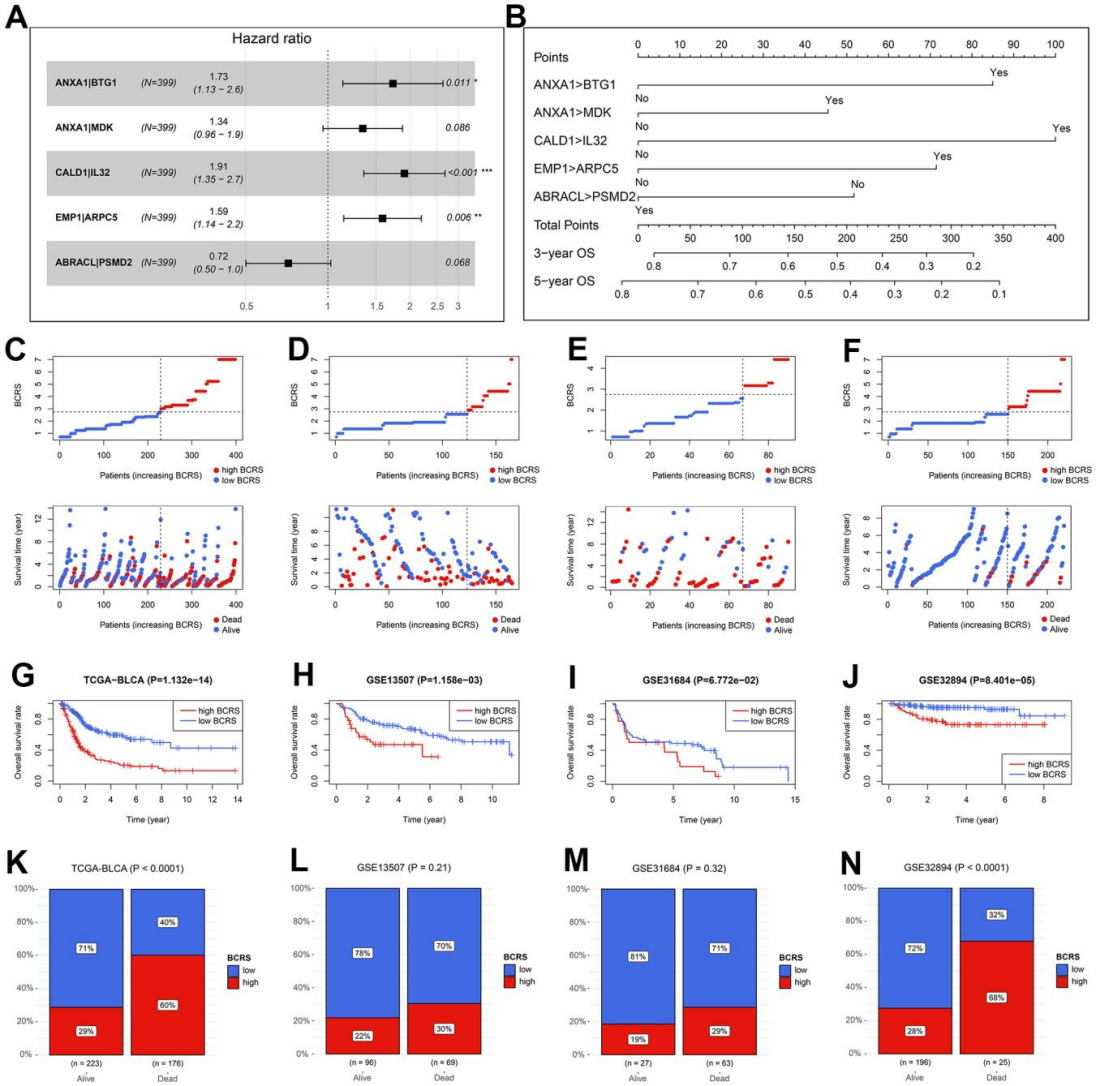
**The validation of the prognosis value of BCRS in BLCA.** (**A**) The hazard ratios and the 95% confidence intervals of the variables in the predictive model. (**B**) A nomogram was drawn to visualize the predictive model. (**C**–**F**) The distribution of the BCRSs and survival statuses of the BLCA cases from the TCGA-BLCA cohort (**C**), the GSE13507 cohort (**D**), the GSE31684 cohort (**E**), and the GSE32894 cohort (**F**). The cut-off values to divide all the subjects in these cohorts into low- and high-BCRS subgroup was equal to 2.75, which was detected by X-tile in the training cohort. (**G**–**J**) The Kaplan-Meier survival analyses of BCRS in the TCGA-BLCA cohort (**G**), the GSE13507 cohort (**H**), the GSE31684 cohort (**I**), and the GSE32894 cohort (**J**). (**K**–**N**) The association between the survival statuses and BCRS stratification in the TCGA-BLCA cohort (**K**), the GSE13507 cohort (**L**), the GSE31684 cohort (**M**), and the GSE32894 cohort (**N**). BCRS, B cell-related score.

The HRs and the 95% confidence intervals (CIs) of the variables in the established Cox proportional hazard model are shown in Figure 5A. To help the researchers and clinicians better understand this model, we established a nomographic chart (Figure 5B), which transformed the complex multiple gene-pair model into a visual graph. Next, we used various public external cohorts to confirm the prognosis value of BCRS. The baseline clinical information of the training and external validation cohorts is presented in Table 1. According to the same cut-off, which equaled 2.75, the subjects from the TCGA-BLCA cohort, GSE13507 cohort, GSE31684 cohort, and GSE32894 cohort were divided into the high- or low-BCRS subgroups. The distribution of the BCRS and the survival statuses of the cases from the TCGA-BLCA cohort, GSE13507 cohort, GSE31684 cohort, and GSE32894 cohort are shown in Figure 5C–5F, respectively. Kaplan-Meier survival analyses displayed the predictive ability of BCRS to OS in the TCGA-BLCA cohort (P < 0.001, Figure 5G), GSE13507 cohort (P < 0.01, Figure 5H), GSE31684 cohort (P > 0.05 Figure 5I), and GSE32894 cohort (P < 0.001, Figure 5J). Besides, more death could be observed in the high-BCRS subjects from these cohorts, as shown in Figure 5K–5N.

**Figure 6:**
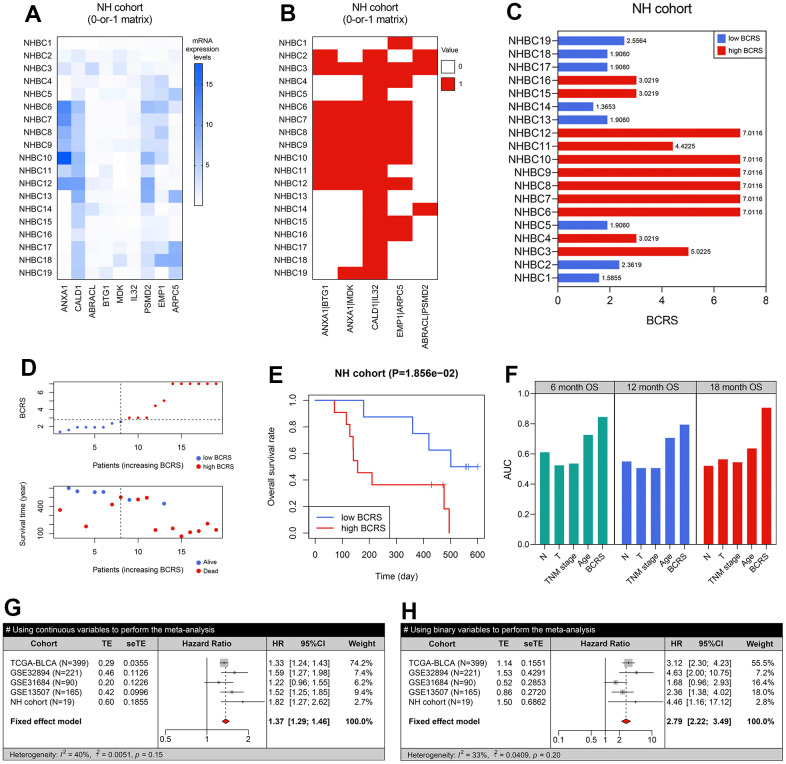
**BCRS was a robust and reliable prognosis predictor in BLCA.** (**A**) The transcriptome expression levels of the model’s genes in the local BLCA samples, which was detected by real-time quantitative PCR. (**B**) A 0-or-1 matrix was established according to the mRNA expression values of the patients. (**C**) The BCRSs of the BLCA samples collected from the local hospital. (**D**) The distribution of survival statuses, follow-up duration, and BCRSs in the NH cohort. According to the same cut-off value (2.75), 19 cases were divided into low- and high-BCRS subgroup. (**E**) The patients with high BCRS exhibited unfavorable OS rates. (**F**) The comparison of BCRS and routine clinicopathological features to 6-. 12-, and 18-months’ OS. (**G**, **H**) Meta-analyses indicated that BCRS was a significant prognosis predictor, both using the continuous variables (**G**) and binary variables (**H**) for analyses.

Besides, 19 BLCA samples collected from the local hospital were used for re-verification. The real-time quantitative PCR experiments were conducted to measure the expression values of the genes included in the model (Figure 6A), and the raw CT levels are shown in Supplementary Table 7. The concrete expression values of the genes and the corresponding clinicopathological information of the patients are supplemented in Supplementary Table 8. Through the RT-qPCR experiments, it was observed that ANXA1, CALD1, EMP1, and PSMD2 were up-regulated in the BLCA samples in comparison to the adjacent normal tissue, while IL32 was down-regulated (all P < 0.05, Supplementary Figure 1A). Additionally, we found that the high expression of ANXA1 was significantly associated with unfavorable prognosis in the NH cohort (P < 0.001, Supplementary Figure 1B). To further clarify the association of ANXA1 with the tumorigenesis of BLCA, cellular experiments were then conducted. Compared with SVHUC1 cells, the T24 (P < 0.05) and UMUC3 (P < 0.01) cells exhibited higher levels of ANXA1 (Supplementary Figure 2).

Using the gene-pair strategy and mRNA expression levels detected by the RT-qPCR experiments, a 0-or-1 matrix of the 5 gene pairs in the predictive model was constructed (Figure 6B). According to the formula mentioned above, the BCRS of each patient was calculated (Figure 6C). The distribution of the survival status, follow-up duration, and BCRSs of the patients is displayed in Figure 6D. The patients in the NH cohort were classified into low- and high-BCRS subgroups based on the cut-off value determined above (2.75), and the cases with high BCRS levels exhibited poorer OS (P < 0.05, Figure 6E). We also compared the prognosis predictive performance of BCRS and routine clinicopathological features to 6-, 12-, and 18-months’ OS, and BCRS showed the highest predictive capability (Figure 6F).

Given that the BCRS was not a significant predictor in the GSE31684 cohort (P > 0.05, Figure 5I), we performed meta-analyses to combine the effects, and the result indicated that BCRS was a significant biomarker for the OS in BLCA, no matter using the continuous variables (pooled HR = 1.37, 95%CI = 1.29-1.46, Figure 6G) and the binary variables (pooled HR = 2.79, 95%CI = 2.22-3.49, Figure 6H).

### BCRS was superior to routine clinicopathological features in OS prediction

The predictive ability of BCRS was compared with the routine clinicopathological parameters, including age, gender, tumor grade, TNM stage, pathological T stage, pathological N stage, and M stage. To ensure comparability, we transformed all the continuous parameters into binary variables. The optimal cut-off for age was determined by X-tile. The univariate and multivariate Cox regression indicated that BCRS was an independent prognosis predictor both in the TCGA-BLCA cohort and the NH cohort (all P < 0.05, Supplementary Table 9). Besides, compared with the common clinical features, BCRS exhibited a stronger predictive ability to 1- (Supplementary Figure 3A), 2- (Supplementary Figure 3B), 3- (Supplementary Figure 3C), 4- (Supplementary Figure 3D), and 5-year’s (Supplementary Figure 3E) OS in the TCGA-BLCA cohort.

### BCRS was a promising tool to evaluate the immunotherapeutic response in BLCA

**Figure 7:**
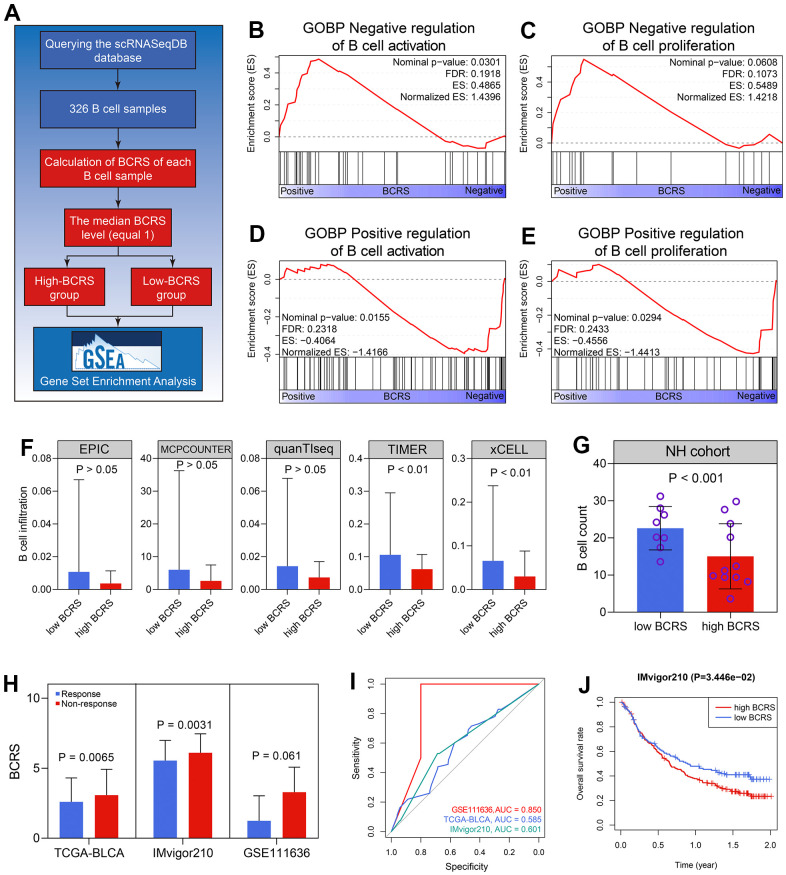
**BCRS was associated with B cells’ activation and proliferation, and served as a potential predictor to immunotherapeutic response.** (**A**) The processes of the GSEA in the 326 B cell samples. (**B**, **C**) The negative regulation of B cells’ activation (**B**) and proliferation (**C**) was enriched in the high-BCRS B cells. (**D**, **E**) The positive regulation of B cells’ activation (**D**) and proliferation (**E**) was enriched in the low-BCRS B cells. (**F**, **G**) BCRS was negatively associated with B cells’ infiltration levels, both in the TCGA-BLCA cohort (**F**) and the NH cohort (**G**). (**H**) The levels of BCRS between immunotherapeutic responders and non-responders from the TCGA-BLCA cohort, IMvigor210 cohort, and GSE111636 cohort. (**I**) The predictive ability of BCRS to immunotherapeutic response in the TCGA-BLCA cohort, IMvigor210 cohort, and GSE111636 cohort. (**J**) High levels of BCRS heralded poor prognosis in the IMvigor210 cohort.

The association between BCRS and B cells’ biological processes was detected based on the 326 B cell samples extracted from the scRNAseqDB (https://bioinfo.uth.edu/scrnaseqdb/). Subsequently, GSEA was performed after the calculation of the BCRS of each B cell sample. The detailed processes are displayed in Figure 7A. Since all the BCRSs of these B cells were less than 2.75, we chose the median BCRS level (equaled 1) to classify the B cell samples into low- and high-BCRS subgroups. It was observed that the negative regulation of B cells’ activation (Nominal P < 0.05, FDR < 0.25, Figure 7B) and proliferation (Nominal P > 0.05, FDR < 0.25, Figure 7C) was enriched in the high-BCRS B cells, while the positive regulation of B cells’ activation (Nominal P < 0.05, FDR < 0.25, Figure 7D) and proliferation (Nominal P < 0.05, FDR < 0.25, Figure 7E) was significant in the low-BCRS B cells, suggesting that BCRS was negatively associated with B cells’ activation and proliferation. In the BLCA patients, the cases with high BCRS exhibited low B cell infiltration levels, both in the TCGA-BLCA cohort (Figure 7F) and the NH cohort (P < 0.001, Figure 7G).

Next, the immunotherapeutic responses of the cases from the TCGA-BLCA cohort were evaluated by the TIDE algorithm. The subjects with low BCRSs were more likely to respond to the immunotherapy through the Wilcoxon signed-rank test (P < 0.01, Figure 7H). Two BLCA cohorts receiving anti-PD1/PDL1 treatment (pembrolizumab for GSE111636 and atezolizumab for IMvigor210) were also utilized for validation in real-world situations. The cases with high BCRSs were less likely to benefit from the atezolizumab treatment (P < 0.01, Figure 7H) in the IMvigor210 cohort. The levels of BCRS in the patients showing response to pembrolizumab were lower than those in the cases exhibiting non-response, but the difference was not statistically significant (P > 0.05 Figure 7H), partly due to the limited sample size. The ROC analyses indicated the predictive ability of BCRS to immunotherapeutic effectiveness in the GSE111636 cohort, IMvigor210cohort, and the TCGA-BLCA cohort (Figure 7I). Besides, in the 195 BLCA patients from the IMvigor210 cohort, the subjects with high BCRS suffered poor prognoses (P < 0.05, Figure 7J). Totally, BCRS was a promising clinical to distinguish the BLCA patients who were more likely to benefit from the immunotherapy.

### Pan-cancer analyses of BCRS

The prognosis value of BCRS at a pan-caner level was also detected. Besides the BLCA, 32 other cancers from the TCGA database were used. Their abbreviations, full terms, and sample size are displayed in Supplementary Table 10. Based on the established formula, the BCRS of each sample was calculated (Supplementary Table 11). It could be observed that BCRSs in different cancers were of high heterogeneity (P < 0.001, Supplementary Figure 4A). The predictive performance of BCRS to DFI, DSS, OS, and PFI in each cancer was then evaluated through univariate Cox regression, and we found that BCRS was a significant prognosis predictor in 21 of 33 cancers (Supplementary Figure 4B and Table 2). At a pan-cancer level, BCRS was negatively associated with B cells’ infiltration proportion in 21 cancer types, and only 6 cancer types showed a positive association (Supplementary Figure 5A). Since tumor mutation burden and microsatellite instability have been widely accepted as biomarkers of immunotherapy, we supplemented the correlation of BCRS to tumor mutation burden (Supplementary Figure 5B) and microsatellite instability (Supplementary Figure 5C) as a reference. Totally, these results implied the tremendous potential of BCRS’s application in other cancer types.

**Table 2:** The prognosis value of BCRS identified by pan-cancer analyses.

**Cancer type**	**BCRS (mean ± SD)**	**OS**		**DFI**		**DSS**		**PFI**	
		**HR (95%CI)**	**P value**	**HR (95%CI)**	**P value**	**HR (95%CI)**	**P value**	**HR (95%CI)**	**P value**
ACC	2.11 ± 1.08	0.28 (0.13-0.61)	0.002	0.14 (0.02-1.12)	0.064	0.26 (0.12-0.59)	0.001	0.38 (0.17-0.86)	0.020
BLCA	2.90 ± 1.80	3.12 (2.29-4.24)	<0.001	2.51 (1.12-5.63)	0.026	2.93 (2.02-4.25)	<0.001	2.02 (1.49-2.72)	<0.001
BRCA	3.03 ± 1.65	1.65 (1.02-2.69)	0.041	2.89 (1.26-6.64)	0.012	1.35 (0.87-2.10)	0.186	1.88 (1.11-3.17)	0.018
CESC	3.07 ± 1.62	1.62 (0.89-2.97)	0.117	0.34 (0.13-0.90)	0.029	1.48 (0.72-3.02)	0.287	0.71 (0.44-1.16)	0.173
CHOL	1.32 ± 0.61	3.99 (0.76-20.95)	0.101	2.43 (0.50-11.87)	0.273	3.82 (0.73-20.06)	0.114	0.51 (0.15-1.74)	0.281
COAD	1.36 ± 0.98	1.43 (0.85-2.41)	0.175	0.26 (0.04-1.96)	0.193	1.64 (0.94-2.86)	0.080	1.54 (1.07-2.22)	0.021
DLBC	1.02 ± 0.22	1.34 (0.32-5.63)	0.692	0 (0-∞)	0.999	0.33 (0.05-2.39)	0.273	1.61 (0.49-5.31)	0.432
ESCA	4.32 ± 2.20	0.62 (0.32-1.18)	0.145	3.47 (0.47-25.91)	0.225	1.59 (0.79-3.21)	0.198	0.78 (0.49-1.24)	0.288
GBM	5.04 ± 1.80	1.3 (0.79-2.14)	0.304	-	-	1.39 (0.80-2.44)	0.247	2.00 (1.12-3.55)	0.018
HNSC	4.94 ± 1.96	1.31 (1.00-1.73)	0.049	1.93 (0.88-4.20)	0.099	1.26 (0.89-1.78)	0.193	1.37 (1.03-1.83)	0.029
KICH	2.71 ± 1.23	0.42 (0.05-3.39)	0.418	7.5 (0.68-83.21)	0.101	1.91 (0.37-9.90)	0.439	1.62 (0.47-5.55)	0.440
KIRC	2.69 ± 1.51	0.46 (0.32-0.66)	<0.001	1.52 (0.54-4.25)	0.428	0.33 (0.22-0.50)	<0.001	0.44 (0.30-0.65)	<0.001
KIRP	2.31 ± 1.15	0.33 (0.16-0.68)	0.003	0.33 (0.14-0.79)	0.013	0.26 (0.12-0.57)	0.001	0.38 (0.20-0.71)	0.003
LAML	1.89 ± 0.54	0.86 (0.43-1.72)	0.664	-	-	-	-	-	-
LGG	3.24 ± 1.69	4.56 (3.12-6.68)	<0.001	1.71 (0.73-4.05)	0.219	4.53 (3.04-6.76)	<0.001	2.73 (2.00-3.75)	<0.001
LIHC	1.20 ± 0.57	1.11 (0.68-1.79)	0.682	1.21 (0.78-1.87)	0.400	0.79 (0.42-1.50)	0.472	1.18 (0.78-1.78)	0.428
LUAD	2.65 ± 1.75	1.42 (0.94-2.15)	0.093	1.88 (1.02-3.47)	0.043	1.37 (0.89-2.12)	0.155	1.40 (0.95-2.06)	0.085
LUSC	4.30 ± 2.15	0.74 (0.54-1.03)	0.074	1.42 (0.84-2.38)	0.188	0.73 (0.45-1.16)	0.181	1.24 (0.72-2.16)	0.438
MESO	4.25 ± 1.59	0.55 (0.34-0.89)	0.014	0.33 (0.06-1.72)	0.189	0.49 (0.27-0.91)	0.024	0.59 (0.35-1.00)	0.051
OV	2.72 ± 1.28	1.96 (1.35-2.85)	<0.001	1.37 (0.97-1.94)	0.073	2.08 (1.42-3.07)	<0.001	1.47 (1.03-2.10)	0.034
PAAD	3.69 ± 2.15	1.62 (1.01-2.58)	0.045	5.42 (1.60-18.35)	0.007	2.1 (1.23-3.59)	0.007	1.88 (1.19-2.98)	0.007
PCPG	2.37 ± 0.90	1.89 (0.47-7.55)	0.370	0.66 (0.07-6.37)	0.721	1.85 (0.37-9.20)	0.451	1.38 (0.59-3.23)	0.457
PRAD	3.15 ± 1.53	2.23 (0.66-7.50)	0.194	0.39 (0.19-0.81)	0.011	0.61 (0.10-3.69)	0.588	0.58 (0.38-0.86)	0.008
READ	1.25 ± 0.79	2.15 (0.91-5.08)	0.080	1.98 (0.35-11.01)	0.437	0.46 (0.16-1.34)	0.155	1.43 (0.63-3.29)	0.393
SARC	3.83 ± 1.80	1.46 (0.97-2.17)	0.066	1.3 (0.81-2.09)	0.278	1.19 (0.77-1.84)	0.432	0.82 (0.59-1.14)	0.246
SKCM	4.47 ± 2.05	1.36 (0.98-1.90)	0.068	-	-	1.47 (1.01-2.13)	0.043	0.83 (0.66-1.04)	0.107
STAD	2.27 ± 1.53	1.42 (1.01-1.98)	0.042	1.68 (0.86-3.27)	0.125	1.75 (1.06-2.87)	0.028	1.6 (1.12-2.29)	0.010
TGCT	1.98 ± 1.37	0 (0-∞)	0.999	1.4 (0.57-3.45)	0.462	3.3 (0.30-36.37)	0.330	2.51 (0.77-8.19)	0.127
THCA	6.17 ± 1.39	0.66 (0.19-2.31)	0.511	0.85 (0.38-1.88)	0.682	0.21 (0.05-0.96)	0.044	0.70 (0.35-1.40)	0.318
THYM	0.98 ± 0.60	5.44 (1.35-21.94)	0.017	-	-	2.79 (0.39-20.08)	0.308	2.34 (0.95-5.76)	0.064
UCEC	1.99 ± 1.13	1.46 (0.96-2.22)	0.073	0.72 (0.39-1.32)	0.289	1.98 (1.12-3.50)	0.020	1.46 (1.00-2.13)	0.049
UCS	2.29 ± 1.18	1.63 (0.70-3.83)	0.258	6.28 (1.39-28.40)	0.017	1.67 (0.73-3.79)	0.222	2.06 (0.80-5.33)	0.135
UVM	3.01 ± 0.80	0.26 (0.09-0.72)	0.009	-	-	0.32 (0.11-0.97)	0.045	0.49 (0.17-1.43)	0.192

BCRS, B cell-related score; SD, standard deviation; OS, overall survival; DFI, disease-free interval; DSS, disease-specific survival; PFI, progression-free interval.

## DISCUSSION

B cells serve as the effector cells of humoral immunity and are essential for T cell immunity during tumor progression [25]. Julius Gordon et al. and Kirk R. Schultz et al. found that the mice with depleted B cells and virus-induced Moloney sarcoma [26] or leukemia [27] tended to exhibit impaired T cell cytotoxic functions, resulting the metastasis and relapse of the tumors. Meanwhile, the negative roles B cells play in tumor immunity also have been widely reported, especially for Bregs. After the injection of Bregs, the tumor proliferation in the human hepatocellular carcinoma model mice has been enhanced [28]. Besides, the depletion of B cells via anti-IgM Ab could inhibit growth and metastases in advanced colon cancer [29]. The conflicting functions of B cells in tumor immunity indicated that B cells might play different roles in different cancers. However, the number of investigations pertaining to B cells in BLCA remains limited.

The present study aims to comprehensively analyze the B cell-related genes’ expression profiles and their association with prognosis and immunotherapy in BLCA. First, the infiltration levels of B cells in the TCGA-BLCA cohort and the local cohort were evaluated through multiple algorithms and CD20 staining, and the results suggested that low B cell infiltration levels heralded unfavorable survival rates. Subsequently, the transcriptome profiles of B cells were compared with other cells in TME at a single-cell level. To screen the B cell-related genes possibly involved in tumorigenesis, we conducted the genomic difference of the B cells’ marker genes between the adjacent normal and BLCA samples extracted from TCGA. Multiple machine learning and computational biology methods, including the gene-pair strategy, LASSO, random forest, and multivariate Cox regression, were performed to generate BCRS. The GEO dataset was comprehensively queried by two independent investigators, and three cohorts were used to validate the prognosis value of the model, and one cohort was obtained to verify the predictive ability of the model for immunotherapy. Importantly, we measured the prognostic value of BCRS in the 19 BLCA patients from our hospital. It should be emphasized that the cut-off value for all the BLCA patients enrolled in this study into low- and high-BCRS subgroups was 2.75. The meta-analyses indicated that BCRS was a reliable predictor for BLCA’s prognosis. 326 B cell samples from the scRNAseqDB indicated that BCRS was negatively associated with B cells’ activation and proliferation, and the TCGA-BLCA cohort and the NH cohort showed that BCRS was negatively associated with B cell infiltration levels. The cohorts receiving anti-PD1/PDL1 and the TIDE algorithm showed that BCRS was a promising tool to evaluate the immunotherapeutic sensitivity in BLCA. At a pan-cancer level, BCRS was a significant prognosis predictor in 21 of 33 cancers, and the negative association of BCRS with B cell infiltration proportion was observed in 21 cancer types.

The rapid development of genomic technologies and big-data analyses provided novel insights into the molecular mechanisms of BLCA. The analyses of the biomarkers from a particular aspect, such as pyroptosis [30], necroptosis [31, 32], autophagy [33, 34], tumor-infiltrating T cells and neutrophils [35], glycolysis [36], and ferroptosis [37], deepened our understandings of the underlying mechanisms and offered the possible therapeutic targets in BLCA. Similar to those excellent researches, our work also helped to uncover the novel molecular mechanisms in BLCA. For instance, despite the fact that ANXA1 has been reported as a BLCA prognostic biomarker in BLCA by several studies [38, 39], which was concurrent with our findings (Supplementary Figure 1), we first uncovered that ANXA1 was negatively associated with B cell infiltration levels in BLCA (Figure 4F–4G).

The limitations of this study should also be stated. First, although 5 independent cohorts and local BLCA samples were used for validation, the present study is inherently limited by its retrospective nature, and a prospective, large-scale, multi-center, and double-blind clinical trial would be helpful to clarify the usefulness of BCRS. Second, the sample size in the local cohort was limited, which caused the possible deviation. Third, we reported some novel B cell-related biomarkers as a prognosis predictor in BLCA, but *in vivo/vitro* experiments to clarify their biological functions in B cells were absent, which should be attempted in the future.

## CONCLUSIONS

In conclusion, a B cell-related gene signature was established to evaluate the prognosis and immunotherapeutic sensitivity, which was externally validated in multiple public datasets and the local cohort, helping to guide the personalized treatment and to provide clues for molecular mechanisms’ exploration in BLCA.

## Supplementary Material

Supplementary Figures

Supplementary Tables 1 and 2

Supplementary Table 3

Supplementary Table 4

Supplementary Table 5

Supplementary Table 6

Supplementary Table 7

Supplementary Table 8

Supplementary Tables 9 and 10

Supplementary Table 11
