# Supplementary material for: Construction and experimental validation of a B cell-related gene signature to predict the prognosis and immunotherapeutic sensitivity in bladder cancer
Source: Aging (Albany NY). 2023 Jun 27;15(12):5355–80. doi: 10.18632/aging.204753 (PMC10333061; doi:10.18632/aging.204753)
Supplement: Supplementary Figures [file aging-15-204753-s001.pdf]

SUPPLEMENTARY FIGURES

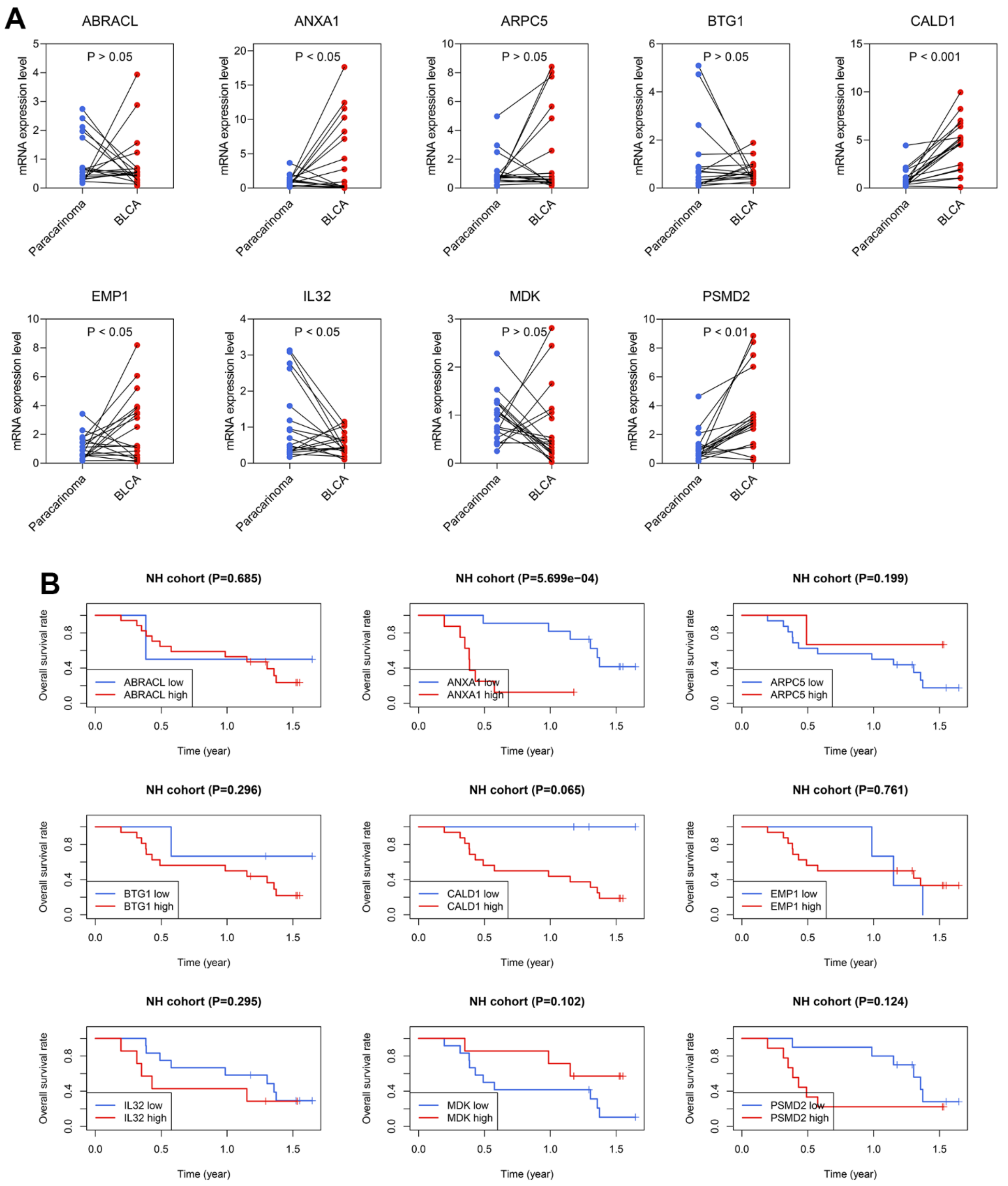

Supplementary Figure 1. The expression difference between adjacent normal and BLCA samples (**A**) and the Kaplan-Meier survival analyses (**B**) of the genes in the established model. The optimal cut-off values to divide the subjects into high- and low-gene expression subgroups were determined by the X-tile software.

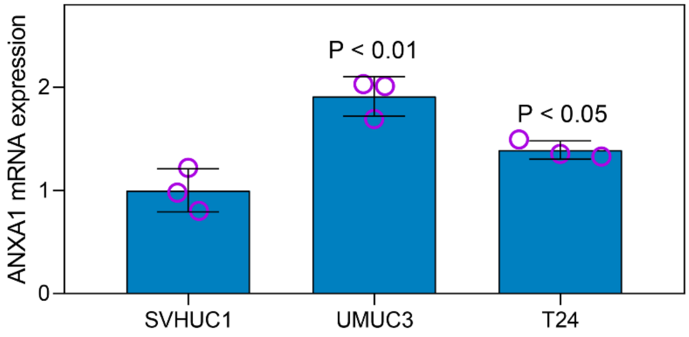

Supplementary Figure 2. The levels of ANXA1 in the SVHUC1, T24, and UMUC3 cells.

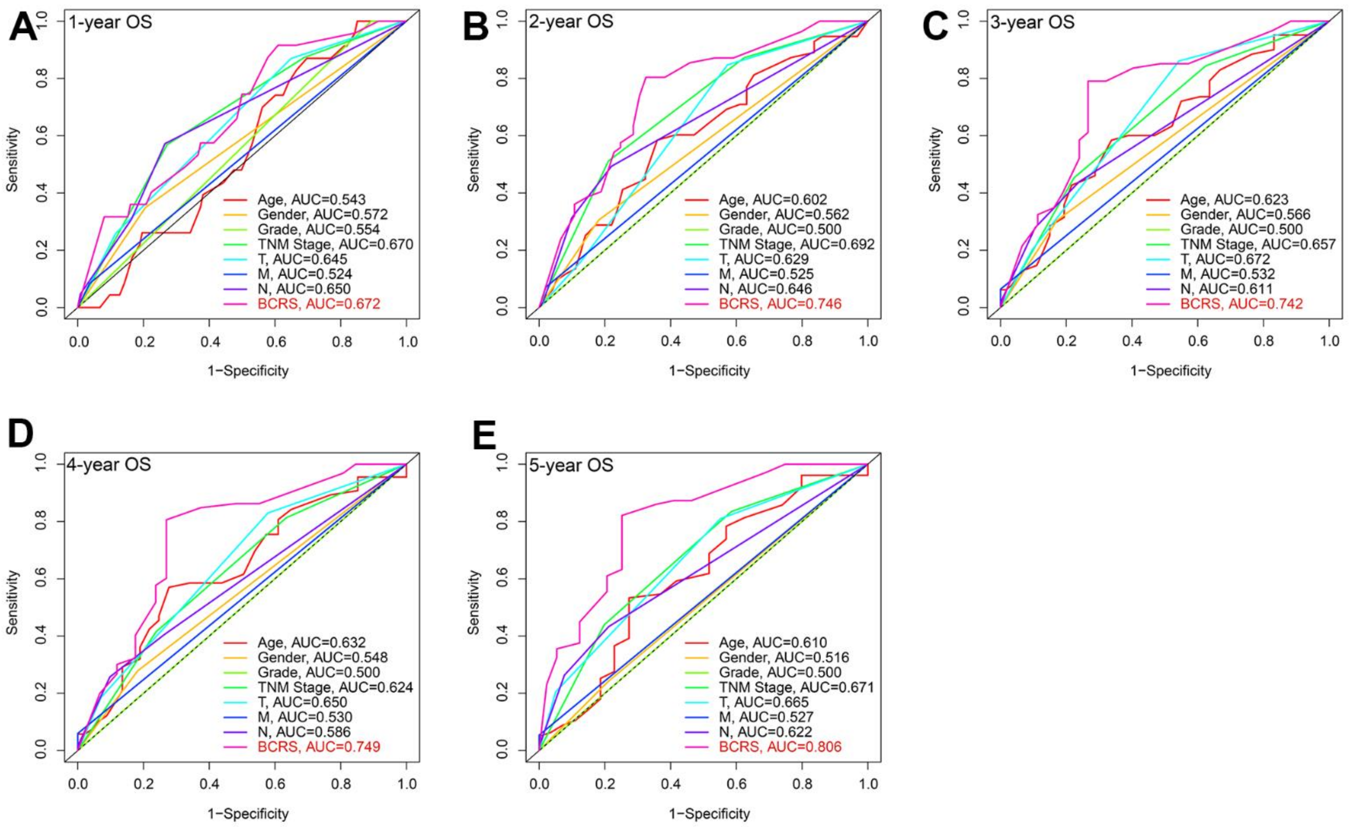

Supplementary Figure 3. The predictive ability of BCRS and the routine clinicopathological features to 1- (**A**), 2- (**B**), 3- (**C**), 4- (**D**), and 5-year's (**E**) overall survival rate in the TCGA-BLCA cohort.

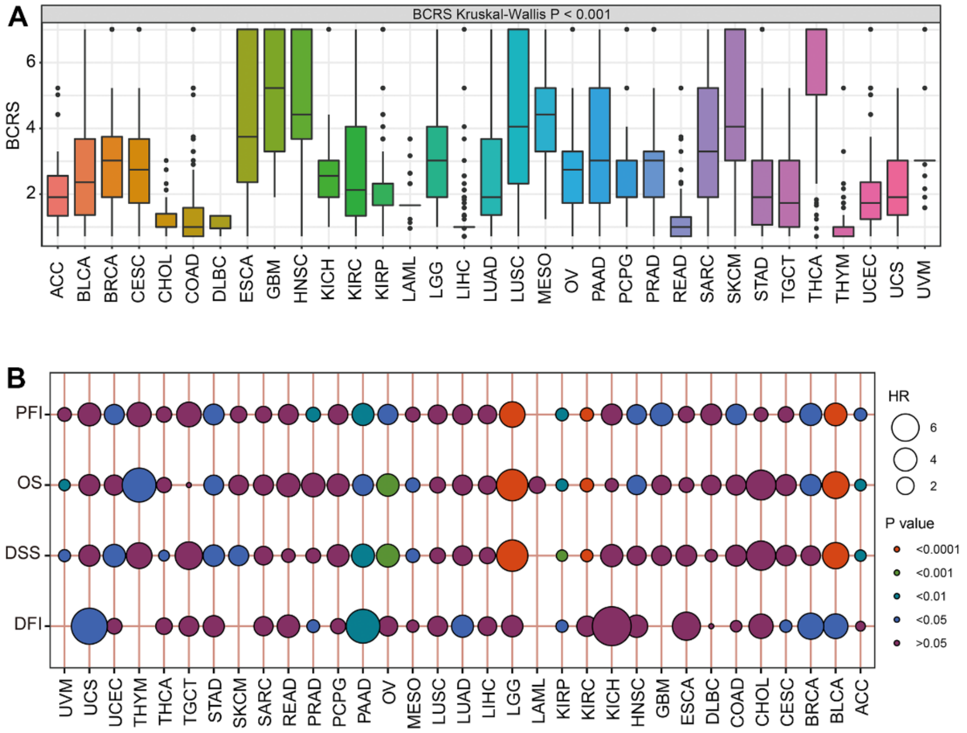

Supplementary Figure 4. Pan-cancer analyses of the prognosis value of BCRS. (**A**) The levels of BCRS in different cancers in TCGA database. (**B**) The predictive ability of BCRS to DFI, DSS, OS, and PFI in 33 cancer types. DFI, disease-free interval; DSS, disease-specific survival; OS, overall survival; PFI, progression-free interval.

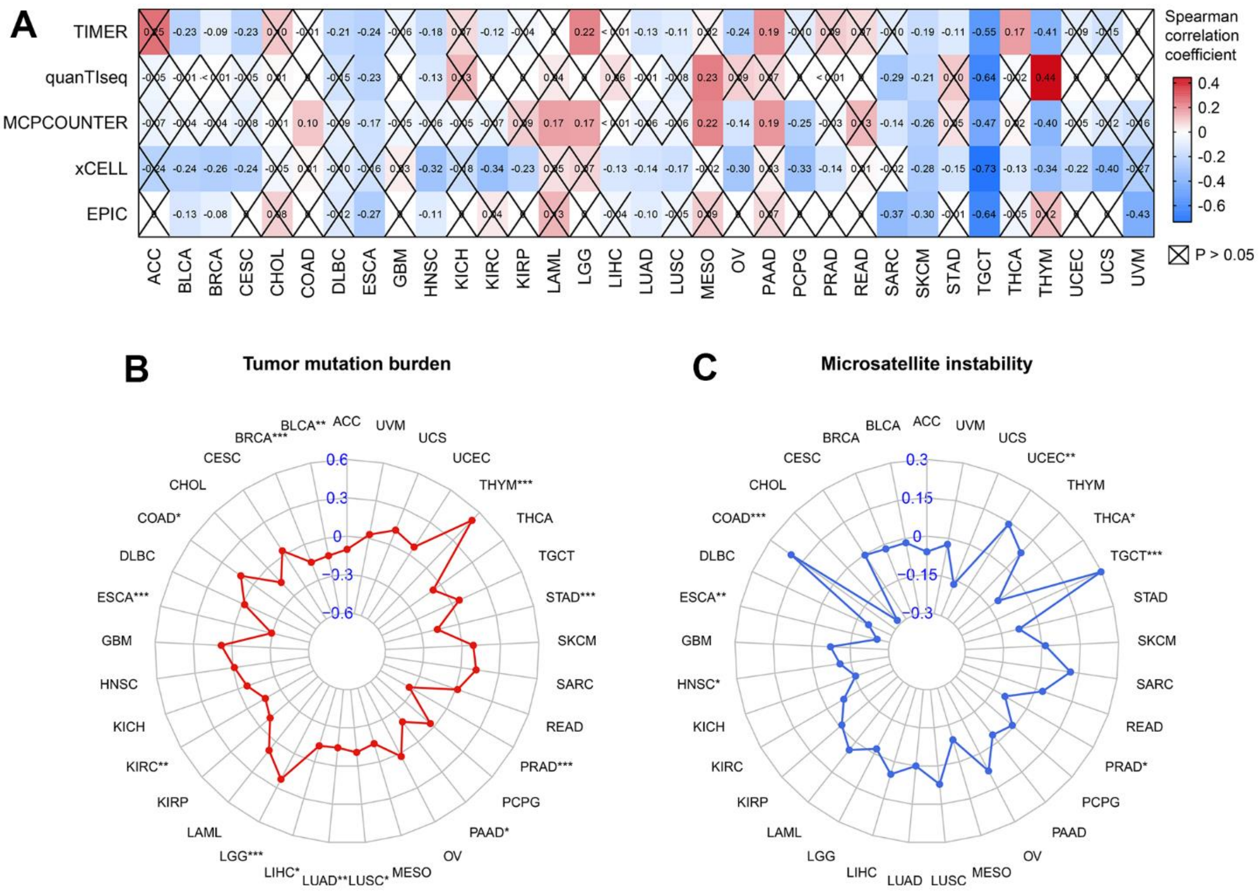

Supplementary Figure 5. Pan-cancer analyses revealing the association of BCRS with B cell infiltration proportion (**A**), tumor mutation burden (**B**), and microsatellite instability (**C**).
