# Supplementary material for: Construction and experimental validation of a B cell-related gene signature to predict the prognosis and immunotherapeutic sensitivity in bladder cancer
Source: Aging (Albany NY). 2023 Jun 27;15(12):5355–80. doi: 10.18632/aging.204753 (PMC10333061; doi:10.18632/aging.204753)
Supplement: Supplementary Tables 1 and 2 [file aging-15-204753-s002.pdf]

SUPPLEMENTARY TABLES

Supplementary Table 1. The detailed information of the public datasets downloaded from GEO.

ID	Experimental type	Platform	Tumor/cell samples	Region
GSE13507	Microarray	GPL6102	165	South Korea
GSE31684	Microarray	GPL570	93	USA
GSE32894	Microarray	GPL6947	308	Sweden
GSE111636	Microarray	GPL17586	11	Spain
GSE145137	Single-cell RNA-seq	GPL16791	2075	South Korea

GEO, gene expression omnibus.

Supplementary Table 2. The primer sequence used in this study.

Gene	Sequence (5'-3')
ANXA1	F: GCGGTGAGCCCCTATCCTA<br>R: TGATGGTTGCTTCATCCACAC
CALD1	F: TGGAGGTGAATGCCCAGAAC<br>R: GAAGGCGTTTTTGGCGTCTTT
EMP1	F: GTGCTGGCTGTGCATTCTTG<br>R: CCGTGGTGATACTGCGTTCC
ABRACL	F: ACCTCTTTGAAGCATTGGTAGG<br>R: GCAGCTCTCCTGGATATGTTAC
BTG1	F: CCACCATGATAGGCGAGATCG<br>R: GGTTGATGCGAATACAACGGTA
MDK	F: CGCGGTCGCCAAAAAGAAAG<br>R: TACTTGCAGTCGGCTCCAAAC
IL32	F: TGGCGGCTTATTATGAGGAGC<br>R: CTCGGCACCGTAATCCATCTC
ARPC5	F: TGGTGTGGATCTCCTAATGAAGT<br>R: CACGAACAATGGACCCTACTC
PSMD2	F: TGCTCGTGGAACGACTAGG<br>R: CAGTTTGCCATAGTGTGGACG
GAPDH	F: GGAGCGAGATCCCTCCAAAAT<br>R: GGCTGTTGTCATACTTCTCATGG
