# Supplementary material for: Construction and experimental validation of a B cell-related gene signature to predict the prognosis and immunotherapeutic sensitivity in bladder cancer
Source: Aging (Albany NY). 2023 Jun 27;15(12):5355–80. doi: 10.18632/aging.204753 (PMC10333061; doi:10.18632/aging.204753)
Supplement: Supplementary Tables 9 and 10 [file aging-15-204753-s009.pdf]

Supplementary Table 9. BCRS is an independent predictor for the OS of BLCA.

Parameters	Univariate Cox		Multivariate Cox	
	**HR (95% CI)**	**P value**	**HR (95% CI)**	**P value**
**TCGA-BLCA cohort**				
Age (<=64 vs. >64)	1.814 (0.976-3.372)	0.06	1.217 (0.634-2.336)	0.556
Gender (Male vs. Female)	1.623 (0.928-2.839)	0.09	1.808 (1.016-3.216)	0.044
Grade (Low vs. High)	2.81E+7 (0-∞)	0.996	1.12e+8 (0-∞)	0.996
TNM Stage (I-II vs. II-IV)	2.499 (1.182-5.285)	0.017	0.629 (0.183-2.162)	0.462
T (T1-2 vs. T3-4)	2.61 (1.278-5.332)	0.008	3.109 (1.014-9.532)	0.047
M (M0 vs. M1)	2.167 (0.779-6.025)	0.138	1.22 (0.411-3.623)	0.721
N (N0 vs. N1-3)	2.312 (1.373-3.891)	0.002	1.776 (0.973-3.241)	0.061
BCRS (Low vs. High)	3.225 (1.891-5.5)	<0.001	3.163 (1.797-5.568)	<0.001
**NH cohort**				
Age (<=64 vs. >64)	0.53 (0.18-1.62)	0.269	0.31 (0.08-1.28)	0.104
TNM Stage (I-II vs. II-IV)	1.34 (0.45-4)	0.603	2.25 (0.41-12.44)	0.352
T (T1-2 vs. T3-4)	1.34 (0.45-4)	0.603	Not available	Not available
N (N0 vs. N1-3)	1.05 (0.32-3.42)	0.937	0.56 (0.07-4.23)	0.573
BCRS (Low vs. High)	4.46 (1.16-17.12)	0.029	6.41 (1.42-28.9)	0.016

Supplementary Table 10. The full name and tumor sample size of the cancer types from TCGA.

Cancer type	Full name	Tumor sample size
ACC	Adrenocortical carcinoma	79
BLCA	Bladder cancer	399
BRCA	Breast invasive carcinoma	1104
CESC	Cervical squamous cell carcinoma and endocervical adenocarcinoma	306
CHOL	Cholangio carcinoma	36
COAD	Colon adenocarcinoma	471
DLBC	Lymphoid neoplasm diffuse large B-cell lymphoma	48
ESCA	Esophageal carcinoma	162
GBM	Glioblastoma multiforme	168
HNSC	Head and neck squamous cell carcinoma	502
KICH	Kidney chromophobe	65
KIRC	Kidney renal clear cell carcinoma	535
KIRP	Kidney renal papillary cell carcinoma	289
LAML	Acute myeloid leukemia	151
LGG	Brain lower grade glioma	529
LIHC	Liver hepatocellular carcinoma	374
LUAD	Lung adenocarcinoma	526
LUSC	Lung squamous cell carcinoma	501
MESO	Mesothelioma	86
OV	Ovarian serous cystadenocarcinoma	379
PAAD	Pancreatic adenocarcinoma	178
PCPG	Pheochromocytoma and paraganglioma	183
PRAD	Prostate adenocarcinoma	499
READ	Rectum adenocarcinoma	167
SARC	Sarcoma	263
SKCM	Skin cutaneous melanoma	471
STAD	Stomach adenocarcinoma	375
TGCT	Testicular germ cell tumors	156
THCA	Thyroid carcinoma	510
THYM	Thymoma	119
UCEC	Uterine corpus endometrial carcinoma	548
UCS	Uterine carcinosarcoma	56
UVM	Uveal melanoma	80
